# Disruption of the *carA* gene in *Pseudomonas syringae* results in reduced fitness and alters motility

**DOI:** 10.1186/s12866-016-0819-z

**Published:** 2016-08-24

**Authors:** Bronwyn G. Butcher, Suma Chakravarthy, Katherine D’Amico, Kari Brossard Stoos, Melanie J. Filiatrault

**Affiliations:** 1 School of Integrative Plant Science, Section of Plant Pathology and Plant-Microbe Biology, Cornell University, Ithaca, NY USA; 2 Emerging Pests and Pathogens Research Unit, Robert W. Holley Center for Agriculture and Health, Agricultural Research Service, United States Department of Agriculture, Ithaca, NY USA; 3 Department of Health Promotion and Physical Education, School of Health Sciences and Human Performance, Ithaca College, Ithaca, NY USA; 4 Present Address: Cornell Lab of Ornithology, Cornell University, 159 Sapsucker Woods Rd, Ithaca, NY USA

**Keywords:** *Pseudomonas syringae* pv *tomato*, *CarAB*, *P32*, Virulence, Swarming, Biofilm formation

## Abstract

**Background:**

*Pseudomonas syringae* infects diverse plant species and is widely used in the study of effector function and the molecular basis of disease. Although the relationship between bacterial metabolism, nutrient acquisition and virulence has attracted increasing attention in bacterial pathology, there is limited knowledge regarding these studies in *Pseudomonas syringae*. The aim of this study was to investigate the function of the *carA* gene and the small RNA *P32*, and characterize the regulation of these transcripts.

**Results:**

Disruption of the *carA* gene (*ΔcarA*) which encodes the predicted small chain of carbamoylphosphate synthetase, resulted in arginine and pyrimidine auxotrophy in *Pseudomonas syringae* pv*. tomato* DC3000. Complementation with the wild type *carA* gene was able to restore growth to wild-type levels in minimal medium. Deletion of the small RNA *P32*, which resides immediately upstream of *carA*, did not result in arginine or pyrimidine auxotrophy. The expression of *carA* was influenced by the concentrations of both arginine and uracil in the medium. When tested for pathogenicity, *ΔcarA* showed reduced fitness in tomato as well as *Arabidopsis* when compared to the wild-type strain. In contrast, mutation of the region encoding *P32* had minimal effect *in planta*. Δ*carA* also exhibited reduced motility and increased biofilm formation, whereas disruption of *P32* had no impact on motility or biofilm formation.

**Conclusions:**

Our data show that *carA* plays an important role in providing arginine and uracil for growth of the bacteria and also influences other factors that are potentially important for growth and survival during infection. Although we find that the small RNA *P32* and *carA* are co-transcribed, *P32* does not play a role in the phenotypes that *carA* is required for, such as motility, cell attachment, and virulence. Additionally, our data suggests that pyrimidines may be limited in the apoplastic space of the plant host tomato.

**Electronic supplementary material:**

The online version of this article (doi:10.1186/s12866-016-0819-z) contains supplementary material, which is available to authorized users.

## Background

The model plant pathogen *Pseudomonas syringae* pv. *tomato* DC3000 (DC3000) infects tomato (*Solanum lycopersicum*) and *Arabidopsis thaliana* (reviewed in [1]). DC3000 enters the apoplastic space through wounds or natural openings in the leaf, like stomata, and grows in intercellular spaces. As the infection progresses, the pathogen releases virulence factors such as the phytotoxin coronatine and injects effector proteins into host cells through the type III secretions system (T3SS). In a susceptible host, chlorosis (yellowing) of the leaves occurs and necrotic lesions develop. Alternatively in a non-host, such as *Nicotiana benthamiana,* a defense-associated hypersensitive response (HR) is elicited.

Most investigations of pathogenicity in *P. syringae* have focused on identifying and characterizing components of the T3SS [2], non-ribosomal peptides [3] and toxins [4, 5]. While these are clearly important, pathogenic bacteria must also compete successfully for limited nutrients within the host, with iron as a well-known example [6]. Unfortunately, it is not well-understood how metabolic processes in plant pathogens contribute to virulence, although experiments using IVET (in vivo expression technology) have identified a variety of bacterial genes expressed during plant-pathogen interactions as well as during host colonization [7–15]. These studies revealed the importance of genes involved in metabolism to the infection process.

Several lines of evidence suggest links between bacterial pathogenicity and metabolism. The disruption of genes involved in acquisition of nutrients such as carbon result in reduced virulence in human and animal pathogens [16–21]. As for plant pathogens, a number of metabolically related genes were identified as required for infection of shoots of apple trees by *Erwinia amylovora* [22] and it was shown that *P. savastanoi pv savastanoi* requires genes directly involved in metabolism in order to survive in olive knots [23]. Arginine metabolism and regulation are associated with the virulence of several pathogenic bacteria such as *Mycobacterium tuberculosis, Listeria monocytogenes, Legionella pneumophila,* and *Mycobacterium bovis* [24–27]. Recently Ramos *et al.* showed that an *argD* mutant in the plant pathogen *Erwinia amylovora* was non-pathogenic [28].

Our laboratory is interested in the identification and characterization of small RNAs in *P. syringae*. Livny *et al.* reported a *Pseudomonas*-specific small RNA (named *P32*) transcribed from an orthologous region upstream from the *carABgreA* operon in *Pseudomonas aeruginosa* [29]. The expression of *P32* was confirmed by Northern blot and a transcript of about 80 bases was detected in rich medium during exponential growth and stationary phase cultures. No other function has been described for this regulatory RNA. This region is also present in the genome of DC3000. While conducting a genome-wide mapping of mRNA 5′ends in DC3000, we identified a potential transcriptional start site 118 bases upstream of *carA* [30]. The *carAB* genes encode the enzyme carbamoylphosphate synthetase (CPSase), which catalyzes the synthesis of carbamoylphosphate, a precursor of arginine and pyrimidines. Further analysis revealed a putative RpoD promoter a short distance upstream from the start site, as well as a potential rho-independent terminator located between the start site and the first codon of *carA*. The promoter may be associated with two overlapping transcripts, a shorter one utilizing the Rho-independent terminator, and a longer one that includes *carA, carB*, and *greA* (pseudomonas.com). Consistent with this model, we observed expression in the region encompassing the 5′UTR, *carA*, *carB*, and *greA* in a transcriptome analysis of the DC3000 genome [31] and detected transcriptional activity in the same region during a search for small RNAs using RNA-Seq (unpublished). Regulation of the *carABgreA* operon in *P. aeruginosa* is controlled both by arginine at the transcriptional level and also by pyrimidines, possibly through an attenuation mechanism [32, 33]. *carAB* mutants of *Pseudomonas* spp. strain G are auxotrophic for arginine as well as pyrimidines [34]. In addition, these mutants are deficient in extracellular polysaccharide production. The function of carbamoyl-phosphate synthase and P32 has not been well characterized in plant pathogenic bacteria. Just recently it was demonstrated that disruption of *carB* in *Xanthomonas citri*, resulted in loss of pathogenicity and inability to elicit a hypersensitive reaction in non-hosts, whereas disruption of *carA* did not affect these phenotypes [35]. However, disruption of *carB* resulted in reduced swimming and reduced ability to form biofilms [36].

The regulation of *P32* as well as *carAB* and their potential contribution to virulence has not been investigated in *P. syringae*. In this study, we investigated *P32* and its involvement in the regulation of *carA* in *P. syringae*. We found that *carA* is important for growth and fitness *in planta* and demonstrated the likely importance of uracil during infection. In contrast, *P32* appears to be involved in *carA* regulation and does not have an obvious role *in planta*, although *P32* is part of the same transcriptional unit as *carA*.

## Results

### Effect of *P32* and *carA* deletions on growth of DC3000

**Fig. 1:**
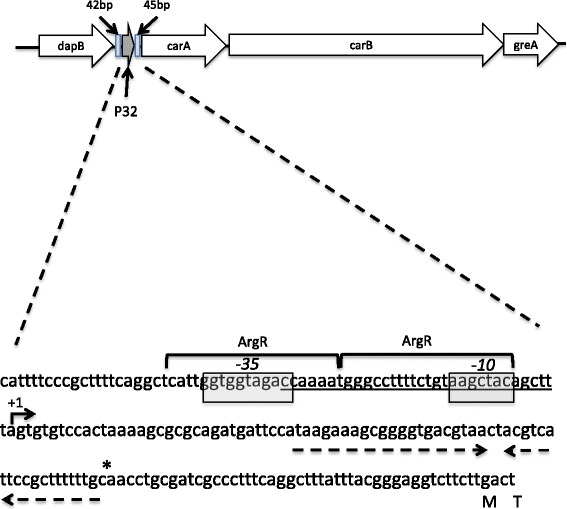
Genomic sequence of the genomic region containing *P32*. The transcriptional start site (reported in Filiatrault et al., 2011) is denoted with an arrow and marked as +1. The putative RpoD-dependent promoter sequence (MEME motif 1;[30]) is underlined. Boxed areas represent the −35 and −10 sites. The ArgR binding sites reported for *Pseudomonas aeruginosa* [42] consisting of two half sites in direct repeat arrangement, is indicated by brackets. A predicted terminator and inverted repeat is denoted by the convergent arrows. The mapped 3' end is denoted by the asterisk. The “M” represents the methionine start codon for CarA and “T” is the symbol for the amino acid threonine. 42 bp denotes the base pairs between the end of the coding region of DapB and the putative ArgR binding site. 45 bp denotes the base pairs from the mapped 3′end of *P32* to the translational start site of CarA

**Fig. 2:**
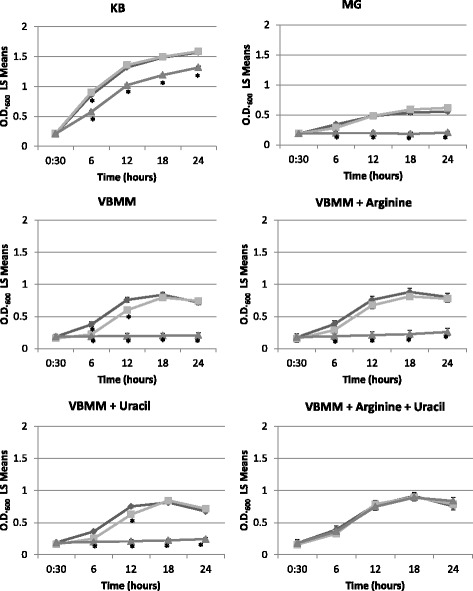
CarA influences growth in minimal medium. Growth of wild type DC3000 (dark gray diamonds), *ΔP32* (light gray squares) and *ΔcarA* (light grey triangles) in KB, MG, VBMM, VBMM supplemented with 40 mM arginine or with 10 mM uracil, and VBMM supplemented with 40 mM arginine and 10 mM uracil. Growth is represented as least squares means with standard error of O.D._600_ over time. The data shown represent three biological replicates per strain, each with three technical replicates. Post hoc comparisons were performed using Tukey HSD (α = 0.05). For each time point, the values which are significantly different from the wild type are shown with an asterisk. Statistical analyses were performed using JMP Pro 11

In previous work, a MEME analysis of DC3000 genomic regions immediately upstream from captured RNA 5′ ends revealed a candidate RpoD promoter adjacent to the putative small RNA P32 [30]. *P32* is located immediately upstream of PSPTO_4502 (*carA*) (Fig. 1). In other organisms the products of *carA* and *carB* are involved in the biosynthesis of arginine and pyrimidines [37] and *carA* mutants have been shown to require arginine for optimal growth [38–41]. We hypothesized that *P32* may also be involved in these pathways since it closely neighbors *carA*. To test the involvement of P32 and CarA in arginine and pyrimidine biosynthesis we constructed two deletion mutants, one in which *P32* was deleted and another in which *carA* was deleted. Deletions were confirmed by PCR and sequencing (data not shown). The transcript for *carA* could still be detected in the *P32* deletion mutant indicating transcription of *carA* can occur in the absence of the genomic region containing *P32* (Additional file 1: Figure S1). Growth of the *P32* deletion mutant was comparable to that of the wild-type strain DC3000 in rich medium KB, minimal medium MG, and minimal medium VBMM (Fig. 2). In contrast, the *carA* mutant displayed a growth defect when grown in rich medium, and minimal media MG and VBMM. The growth defect was abolished by the simultaneous addition of arginine and uracil to VBMM. Also, complementation of the *carA* mutant by expressing the coding region of *carA* on a plasmid, restored growth to wild type levels (Additional file 1: Figure S2), indicating that the *carA* gene was solely responsible for the phenotype observed. Overall the data suggests that *P32* is not required for expression of *carA* and that *carA* is involved in metabolism of arginine and uracil.

### Expression of *P32* and *carA* in DC3000

In *P. aeruginosa*, the expression of *carA* is controlled by both pyrimidines and arginine [32, 33]. To investigate if *P32* influences the expression of *carA* in *P. syringae*, we performed a series of transcriptional analyses. First, to confirm transcriptional activity and to verify the 3′ end of *P32* we performed 3′ RACE. A 3′ end was identified at nucleotide position 5073275c, immediately downstream from the predicted Rho-independent terminator (Fig. 1). The presence of a 3′end in this region could occur as a result of several events. One possibility is that distinct promoters produce two separate transcripts, one containing *P32* and another containing *carA, carB*, and *greA*. Alternatively a transcript could arise from a single transcriptional start site, but under certain conditions termination or cleavage/processing could result in the generation of a small transcript containing *P32* alone. Our 5′end mapping data did not detect another transcriptional start site for *carA* [30] although our transciptome survey detected expression through the entire *P32-carA-carB-greA* operon [31]. A second 5′ mapping experiment using fluorescently labeled oligonucleotide extension (FLOE) detected a single transcriptional start site using RNA isolated from cells grown in VBMM and VBMM supplemented with arginine and uracil (data not shown). This suggests that expression of the entire *P32 –carA* region may be under the control of a single promoter, as is the case in *P. aeruginosa* [32, 33].

**Fig. 3:**
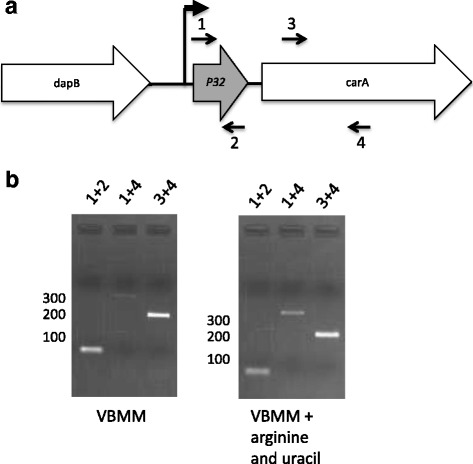
Co-transcription of *P32* and *carA*. **a** Map of the genomic region containing *dapB*, *P32* and *carA* in DC3000. The locations and orientations of RT- and PCR primers are indicated. **b** Agarose gel electrophoresis result of the RT-PCR experiments using the primers pairs indicated. The expected length of the PCR products for the primer pairs are as follows: Primer pair 1 and 2, ~54 bps; primer pair 1 and 4, ~370 bps; and primer pair 3 and 4, ~209 bps. Control reactions in which reverse transcriptase was omitted were performed for each primer set and RNA sample

To investigate if *P32* is co-transcribed with *carA,* RNA was isolated from cells grown under rich or growth-limiting conditions. cDNA synthesis was performed and used for bridging PCR with different primer pairs to identify RNA that consists of both *P32* and *carA* (see Fig. 3a). Products were obtained with each primer set using RNA from bacteria grown with or without arginine and uracil (Fig. 3b). This suggests that *P32* and *carA* are co-transcribed under the conditions tested. These data also indicate that *carA* is transcribed even in the presence of arginine and uracil.

**Fig. 4:**
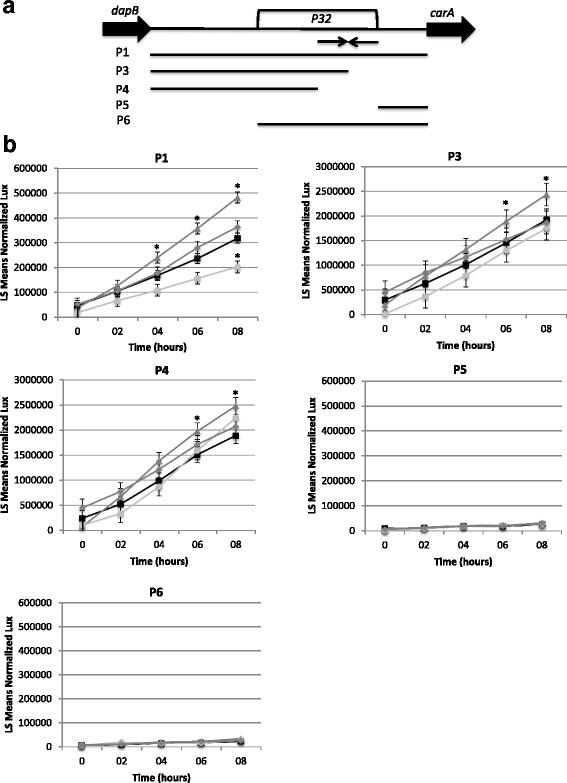
Expression of *P32* and *carA*. **a** Regions of varying lengths upstream to the *carA* coding region were cloned into *lux* reporter constructs; the regions they span are shown graphically. The inverted arrows represent the predicted stem-loop of the Rho-independent terminator. (**b**) Expression from *lux* promoter fusions were evaluated in VBMM medium (black squares), VBMM supplemented with 40 mM arginine (dark gray triangles), VBMM supplemented with 10 mM uracil (light gray circles), and VBMM supplemented with 40 mM arginine and 10 mM uracil (dark gray diamonds). Data shown are the least squares means (LS Means) with standard error of normalized luminescence (lux) values over time, derived from at least 3 independent biological replicates for each promoter fusion-medium combination, each containing 2–3 technical replicates. Note that the scales for each panel are different in order to clearly show statistically different data points. For each time point, the values which are significantly different from VBMM are shown with an asterisk (using Tukey HSD, α = 0.05). Normalized luminescence (lux) is the ratio of luminescence to OD_600_. Statistical analysis was performed using the program JMP Pro11

To further investigate the regulation of *P32* and *carA* we created promoter fusions and evaluated their expression in the wild-type strain (Fig. 4, top panel). The promoter fusions consisted of either the entire intergenic region between *dapB* (PSPTO_4503) and *carA*, including *P32* (referred to as P1), the region from the 3′ end of *dapB* to the first half of the Rho-independent terminator, which therefore lacks ½ of the stem-loop (referred to as P3), the region from the 3′end of *dapB* to the beginning of the stem-loop (lacks the entire stem-loop and all sequence downstream; referred to as P4), *P32* and downstream sequences up to *carA* (lacks putative promoter sequence; referred to as P5), or the region between the 3′ end of *P32* and *carA* (lacks putative promoter region and *P32*; P6). Fusions lacking the putative promoter region (P5 and P6) were expressed at background levels in VBMM or VBMM supplemented with arginine or uracil (Fig. 4). This is consistent with the single mapped transcriptional start site for *carA* and *P32* and the co-expression data that indicates *P32* and *carA* are transcribed together from a single promoter under these conditions. In addition, we observed an increase in expression when the stem-loop structure was disrupted (P3) or completely removed (P4) compared to the full-length fusion P1. We conclude that this feature is important in modulating the expression of *carA*.

Interestingly, when arginine was added to the medium we observed an increase in expression from promoter fusions P1, P3 and P4 (Fig. 4). The addition of uracil resulted in a decrease in expression of *lux* from the promoter fusions P1. The addition of both arginine and uracil had little effect on the expression of the promoter fusions.

To further investigate the expression of *P32* and *carA*, qRT-PCR was performed with RNA isolated from wild-type cells grown in VBMM and VBMM supplemented with 40 mM arginine and 10 mM uracil. Although transcripts for *P32* and *carA* were detected in both growth conditions, no difference in expression was observed between cells grown in VBMM or VBMM supplemented with arginine and uracil (data not shown). This is consistent with the promoter fusion data.

### ArgR regulates expression of *P32* and *carA*

**Fig. 5:**
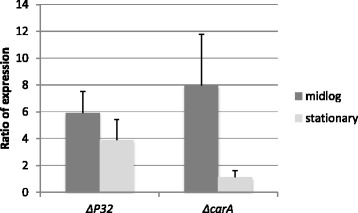
Expression of *P32* and *carA* in wild-type DC3000 compared to *ΔargR* mutant using qRT-PCR. The dark gray bars represent the ratios of the transcripts comparing *ΔargR* mutant to the WT at mid-log phase, and the light gray bars represent the ratios of the transcripts comparing Δ*argR* mutant to the WT at stationary phase. RNA samples were normalized using *gap1*. The Δ*argR* mutant shows increased levels of *P32* and *carA* transcript compared to the WT at mid-log phase. The levels of *P32* and *carA* transcripts were analyzed by calculating the fold difference of transcript levels between WT and Δ*argR* mutant using the Δ C_t_ method. Data shown are the average and standard deviation of three independent biological replicates

ArgR binds to a region upstream of *carA* in *P. aeruginosa* [33, 42]. Although ArgR can act as a repressor or activator, in *P. aeruginosa* it has been shown to act as a repressor of *carA* expression [42]. Because the predicted binding site for ArgR, TGTCGCN_8_AAN_5_ appears to be conserved in *P. syringae* (Fig. 1), we hypothesized that ArgR would also regulate *P32* and/or *carA* in *P. syringae*. We analyzed the expression levels of *P32* and *carA* in the *ΔargR* mutant and wild-type DC3000 using qRT-PCR. Our results show that expression of *P32* is increased in the *ΔargR* mutant compared to the wild-type strain at mid-log and stationary phases, while *carA* expression is increased during mid-log phase (Fig. 5). These observations indicate that ArgR likely acts as a repressor of *P32* and *carA* in DC3000.

### Examining the contribution of *P32* and *carA* to virulence

**Fig. 6:**
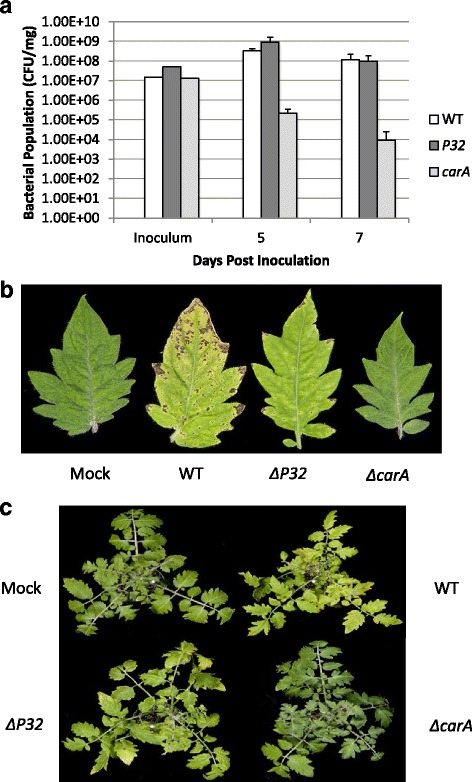
*carA* contributes to fitness in tomato. **a** Four-week-old Tomato cv. MoneyMaker tomato plants were dipped in suspensions containing 1 × 10^7^ CFU ml^−1^ of WT, *ΔP32*, or *ΔcarA*. At the time points indicated, bacteria were extracted from leaves and plated on KB containing rifampicin for enumeration. The values shown are the average CFU/mg with standard deviation from three plants per strain. Similar results were obtained in two repetitions of the experiment. **b** Tomato leaves photographed at 7 dpi (**c**) Whole tomato plants photographed at 7 dpi

To test the involvement of P32 and CarA in virulence, tomato plants were dipped in suspensions of wild-type, Δ*P32* mutant, and the Δ*carA* mutant. The Δ*carA* mutant displayed less intense disease symptoms and reduced bacterial growth on days 5 and 7 post-inoculation compared to the wild type (Fig. 6). Although Δ*P32* growth was similar to wild- type, it displayed reduced symptoms (Fig. 6). However, the symptoms caused by the Δ*P32* mutant were more intense than Δ*carA* mutant.

**Fig. 7:**
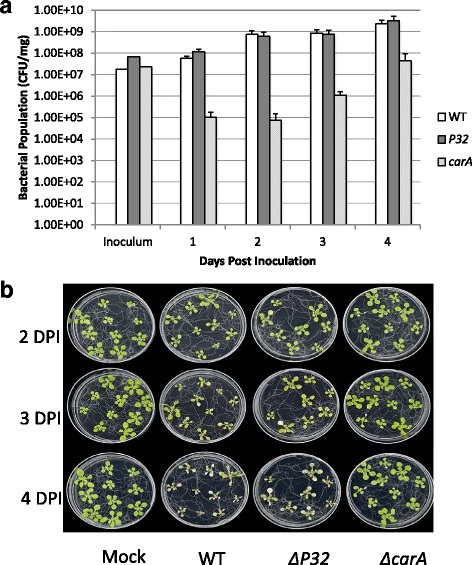
*carA* contributes to fitness in Arabidopsis seedlings. **a** Arabidopsis seedlings were inoculated with suspensions containing 1 × 10^7^ CFU ml^−1^ of WT, *ΔP32*, or *ΔcarA*. At the time points shown, bacteria were extracted from leaves and plated on KB containing rifampicin for enumeration. The values shown are the average CFU/mg with standard deviation of three seedlings per strain. The experiment was repeated twice with similar results. **b** Disease phenotype of *Arabidopsis* seedlings flood-inoculated with a bacterial suspension of WT, *ΔP32*, or *ΔcarA*. Mock-inoculated seedlings were flooded with sterile distilled H_2_O containing 0.025 % Silwet L-77. Photographs were taken 2, 3, and 4 dpi

Since the Δ*carA* mutant had reduced virulence in tomato, we tested the ability of this mutant and *ΔP32* to cause disease in *A. thaliana* seedlings. *ΔP32* grew to similar levels as the wild type (Fig. 7a). In addition, the chlorotic symptoms caused by *ΔP32* were also similar to wild type (Fig. 7b). Based on these data, it is unlikely that Δ*P32* plays a substantial role in virulence in DC3000. However, the Δ*carA* mutant displayed reduced growth and was not able to cause the same necrotic symptoms as the WT (Fig. 7a and b), suggesting that *carA* is necessary for growth and fitness *in planta*.

### Growth of *ΔP32* and *ΔcarA* in apoplastic fluid

**Fig. 8:**
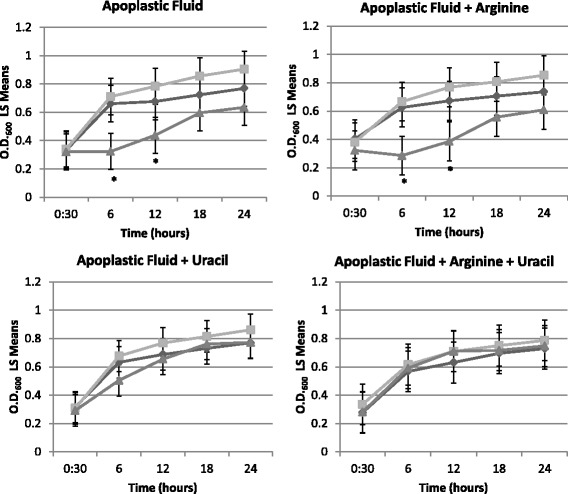
CarA contributes to growth in apoplastic fluid. Growth of wild type DC3000 (dark gray diamonds), *ΔP32* (light gray squares) and *ΔcarA* (light grey triangles) in apoplastic fluid, apoplastic fluid supplemented with 40 mM arginine or 10 mM uracil, and apoplastic fluid supplemented with 40 mM arginine and 10 mM uracil. Growth is represented as least squares means (LS Means) with standard error of O.D._600_ over time. The data shown represent 3 biological replicates per strain, each with 3 technical replicates. Post hoc comparisons were performed using Tukey HSD (α = 0.05). For each time point, the values which are significantly different from wild-type are shown with an asterisk. Statistical analyses were performed using JMP Pro 11

During infection, *P. syringae* obtains its nutrients from the apoplast. Therefore to investigate whether the observed reduction in growth *in planta* was due to nutrient limitation, we compared the growth of wild-type DC3000, *ΔP32,* and *ΔcarA* in apoplastic fluid extracts. The wild-type strain and the *ΔP32* mutant demonstrated similar growth. However, growth of *ΔcarA* was lower than the wild-type at earlier time points in apoplastic fluid with or without arginine. However, *ΔcarA* was able to achieve growth levels similar to wild type at later time points (Fig. 8). In apoplastic fluid supplemented with uracil or both arginine and uracil, *ΔcarA* and *ΔP32* growth characteristics were similar to wild type, with no significant differences detected between the strains at all time points (Fig. 8).

### *ΔcarA* is reduced in motility

**Fig. 9:**
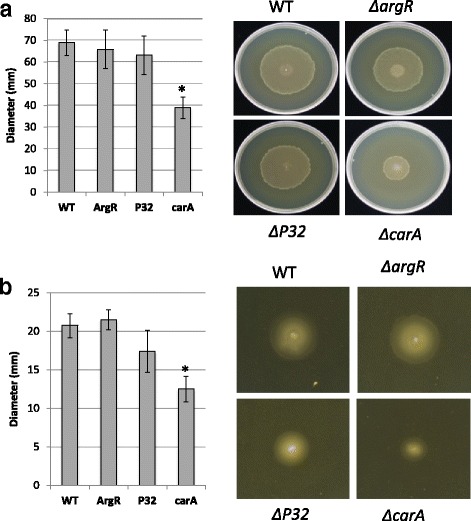
Disruption of *carA* impairs motility. **a** Swarming of *Pseudomonas syringae* DC3000, Δ*argR*, *ΔP32*, and *ΔcarA* after 24 h. **b** Diameter of swimming colonies of *Pseudomonas syringae* DC3000, Δ*argR, ΔP32*, and *ΔcarA* after 24 h. The error bars represent the standard deviation of the mean. Data were analyzed by one-way analysis of variance (ANOVA) followed by Tukey HSD for pair-wise comparisons. Asterisks indicates significant difference for swarming (ANOVA, *P*-value <0.004 and Tukey HSD, *P*-values <0.01) and swimming (*P*-value < 0.002 using ANOVA; *P*-values <0.05 using Tukey HSD)

The reduced virulence observed with the *ΔcarA* mutant could be solely due to the inability to grow in vivo or the inability to produce other factors related to virulence. Because *carA* is induced in *Salmonella* cells that are swarming compared to cells that are in a vegetative state, and has been implicated in motility [43], we tested the ability of the *ΔcarA* mutant to swarm. Since the *ΔcarA* mutant does not grow as efficiently as the wild-type strain in minimal media, the assay was conducted in nutrient agar, a rich medium that is used to test swarming of *P. aeruginosa*. All strains grew equally in this medium (data not shown). As shown in Fig. 9a, the wild-type strain, *ΔargR* mutant and *ΔP32* mutant swarmed equally (Fig. 9a) in this medium while the *ΔcarA* mutant exhibited reduced swarming (ANOVA, *P*-value <0.004 and Tukey HSD, *P*-values <0.01) (Fig. 9a). Motility was also examined using a soft agar to test for functional flagella. The *ΔcarA* mutant showed reduced swimming compared to the wild-type, *ΔargR* mutant and *ΔP32* mutant (ANOVA *P*-value < 0.002; Tukey HSD *P*-value <0.05) (Fig. 9b).

### Deletion of *carA* affects cell attachment

**Fig. 10:**
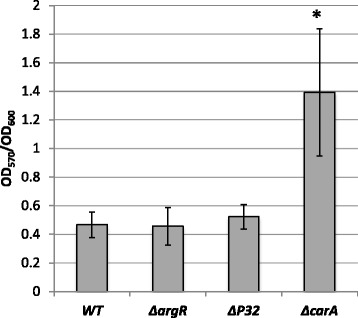
Disruption of carA enhances biofilm formation. Biofilm formation by *P. syringae* DC3000, Δ*argR, ΔP32*, and *ΔcarA*. Cells were grown for 72 h at 28 °C in 96-well microtiter plates containing nutrient broth, and surface-associated biofilm formation was analyzed by crystal violet staining of the adherent biofilm, extraction of the crystal violet with acetic acid, and measurement of the absorbance (OD_570_). All experiments were done in triplicate with at minimum of three technical repeats. Data were analyzed by one-way analysis of variance (ANOVA) followed by Tukey HSD for pair-wise comparisons. Asterisks indicate statistically significant difference (*P* < 0.003 by ANOVA; *P* < 0.01 using Tukey HSD)

Since motility plays an important role in the ability of the bacteria to colonize different environments and attach to surfaces, we examined the Δ*carA* mutant using the microtiter dish assay that has become a standard tool for the study of the early stages in biofilm formation [44]. The Δ*carA* mutant showed a statistically significant (*P* < 0.003 by ANOVA; *P* < 0.01 using Tukey HSD) increase in biofilm formation in comparison to the wild type (Fig. 10). No observable growth differences were observed when the OD_600_ of planktonic cells was measured as a function of time during the period of growth in the microtiter wells.

## Discussion

Although the *carAB* operon is conserved in many bacteria, its regulation is surprisingly variable. [37]. The well-characterized *carAB* operon in *E. coli* (reviewed by [45]) is regulated by several mechanisms. This operon makes use of two tandem promoters that are separately regulated by pyrimidines and arginine. The more distal or upstream promoter is regulated by numerous factors including integration host factor (IHF), the purine repressor (PurR), the pyrimidine ultilization regulator (RutR) as well as PepA, an aminopeptidase and a UMP-kinase PyrH. Additional regulation occurs through reiterative transcription (or RNA polymerase stuttering) when dUTP is available at high concentration, nascent transcripts originating at this promoter are released prematurely due to RNA polymerase stuttering at a T-rich region immediately downstream from the transcriptional start site. The second (proximal) promoter is negatively regulated by the transcriptional regulator ArgR which consists of two trimers that are stabilized by the binding of arginine.

In contrast to *E.coli*, *carAB* in *P. aeruginosa* is transcribed from a single promoter [32]. *carAB* expression increases in response to limitation of either arginine or pyrimidine. The *carAB* transcript includes an upstream untranslated region (UTR) that contains a potential stem-loop structure [32]. The pyrimidine response is reduced when a portion of the right arm of the stem-loop structure is deleted, and it is abolished when the stem-loop structure is completely removed. Since *carAB* expression continues to be responsive to arginine levels in these experiments, the stem-loop structure appears to be required specifically for pyrimidine regulation of *carAB.*

Our data shows that regulation of *carAB* in DC3000 resembles the regulation reported in *P. aeruginosa.* However there are some novel features. Although the qRT-PCR data suggests that ArgR represses the expression of *P32* and *carA*, unexpectedly our promoter fusion data shows that addition of arginine to the medium increases the expression of *P32* and *carA* in contrast to the repression observed in *P. aeruginosa*. However the regulation of the pyrimidine pathway in *Pseudomonas* is strongly influenced by pyrimidine and purine nucleotide effectors [46]. For example, the activity of the carbamoyl-phosphate synthase is inhibited by UMP and activated by ornithine and N-acetylornithine [47] and *carAB* expression is subject to pyrimidine control via an attenuation mechanism. Therefore it is possible, that under the conditions we examined expression, the DC3000 cells are experiencing a high requirement for pyrimidines and expression of *P32* and *carA* is not repressed upon supplementation of arginine. Additionally, the CPase of previously studied pseudomonads shows in most cases only limited repression by arginine and the ability of arginine to repress genes involved in arginine biosynthesis is sometimes influenced by carbon source [37]. Taken together our data indicates that the regulation of *P32* and *carA* is complex in *P. syringae* and differs from the regulation observed in *P. aeruginosa*. More extensive analyses are needed to determine direct regulation by ArgR and further characterize possible post-transcriptional regulation that may be occurring in these pathways in *P. syringae*.

Another difference we observed when compared to the *P. aeruginosa* is in the 5′UTR of *carA*. Our unpublished data of sRNAs found in *P. syringae* along with the mapping of the 3′ end in this study supports the notion that a small transcript is produced from the 5′UTR of *carA*. Interestingly, in *P. aeruginosa* a leader peptide is produced from the *carA* promoter region [32]. Inspection of the *P. syringae* DC3000 *carA* promoter region did not reveal a possible start codon that could give rise to a small peptide in this region (data not shown). Recently it was predicted that in *P. syringae* pv. *phaseolicola* 1448A *carA* is regulated by attenuation [48]. We hypothesize that in DC3000 *P32* is generated by transcription attenuation. A sRNA derived from the 5′UTR of *carA* might act *in trans* to regulate expression of other genes. This concept was first described in *E. coli* [49]. Recent studies have shown that 5′UTRs of pathogenic bacteria can accumulate as stable RNA molecules [50] and are capable of acting *in trans*. Work in *L. monocytogenes* showed that several *cis*-acting riboswitches located in the 5′ UTRs of mRNAs produce small transcripts as the result of premature transcription and these target and regulate the expression of other mRNAs *in trans* [51]. The possibility that P32 may act *in trans* is intriguing. Since this sRNA is conserved among the Pseudomonads, this could add a new complexity to the regulation of arginine biosynthesis in the Pseudomonads and could identify regulatory links between arginine and other regulatory pathways in these bacteria.

We found that in *P. syringae* a *carA* mutant displays reduced growth in apoloplastic fluid and reduced fitness *in planta*. During a screen for DC3000 mutants that displayed reduced virulence, Brooks, D.M. *et al*. discovered that a mutant with a Tn5 insertion in the *carA* gene showed reduced virulence in *A. thaliana* [52]. Although the authors suggested that the inability of the mutant to multiply to high levels in *A. thaliana* leaves was likely because of limited nutrients in the apoplast of *A. thaliana* leaves, no further studies were performed. Interestingly our studies have shown that the growth defect of *ΔcarA* could not be restored at 6 or 12 h with addition of arginine to apoplastic fluid. Surprisingly, growth of *ΔcarA* at the earlier time points could be restored to wild-type levels with the sole addition of uracil suggesting the supply of pyrimidines may be a limiting growth factor in apoplastic fluid. These data imply that there maybe sufficient arginine concentrations *in planta* but pyrimidines may be limiting thus resulting in reduced fitness *in planta*. Studies have shown that of the 20 protein amino acids, arginine was the only amino acid that could not be detected in apoplastic fluid [53]. To our knowledge the concentrations of pyrimidines in the tomato hosts have not been reported. It has been reported that *Erwinia amylovora* can obtain sufficient pyrimidines from host tissue to support growth and cause disease [54]. The situation we observe with *P. syringae* is more similar to the findings reported for some human bacterial pathogens, where *de novo* pyrimidine synthesis is required for growth in host-derived material [55].

The *P32* mutant was able to grow to wild type levels *in planta* and in apoplastic fluid extracts. However, it caused reduced disease symptoms in tomato. Previous studies using DC3000 mutants have shown that reduced symptom formation is not always associated with reduced growth *in planta* [56]. The precise role of *P32* in as yet undefined regulatory pathways that may lead to symptom production needs to be examined further.

The *carA* mutant formed better biofilms but was also compromised in its ability to swarm. Several mutants with insertions within genes involved in the pyrimidine nucleotide biosynthetic pathway and arginine metabolism displayed reduced biofilm formation [57, 58]. In *Vibrio parahaemolyticus* [59] a *carA* transposon mutant forms only thin pellicles at the air–medium interface. The involvement of *carA* in biofilm formation and swarming of *P. syringae* suggests that the reduced fitness *in planta* may be the result of multiple factors.

*carAB* mutants of *Pseudomonas* spp. strain G are auxotrophic for arginine as well as pyrimidines but also deficient in several traits [34] such as extracellular polysaccharide production. Interestingly, the *carAB* genes from *Pseudomonas* sp. strain G are required for the degradation of diffusible signal factor (DSF), a fatty acid signal molecule involved in regulation of virulence in several *Xanthomonas* species as well as *Xylella fastidiosa* [34]. Interestingly, a *carAB* mutant strain of *Halomonas eurihalina* is also deficient in exopolysaccharide production [41]. This deficiency is thought to be a result of a decrease in the UDP-sugar pool. These compounds are essential to the synthesis of nucleotide di-phospho-sugar precursors such as UDP glucose and UDP galactose. UDP sugar is utilized in the synthesis not only of extracellular polysaccharides but also of lipopolysaccharides and the glycosylation of lipids and fatty acids. It is possible that the *carA* mutant of *P. syringae* displays altered production of extracellular polysaccharides. At least three exopolysaccharides (Psl, Pel, and alginate) contribute to biofilm formation in *P. aeruginosa* [60]. *P. syringae* DC3000 is able to produce Psl and alginate but does not encode for genes for the polysaccharide Pel [61]. Alterations in production of Psl can influence biofilm formation and swarming motility of *P. aeruginosa* [62]. Our future studies will explore if there is an involvement of *carA* in exopolysaccharide production in *P. syringae*.

## Conclusions

In this study we found that *carA* of *P. syringae* plays an important role in providing arginine and uracil for growth of the bacterium and also influences other factors that are potentially important for growth and survival during infection. In conclusion, our data also show that *carA* is important for growth and survival of *P. syringae in planta*.

## Methods

### Bacterial strains and growth conditions

The bacterial strains and plasmids used in this study can be found in Additional file 2: Table S1. *Pseudomonas syringae pv. tomato* DC3000 (DC3000) was cultured at 28 °C or at room temperature on King’s B (KB) agar [63]. Where noted, the minimal media used were MG Mannitol-Glutamate (MG) medium (10 g/L of mannitol, 2 g/L of L-glutamic acid, 0.5 g/L of KH_2_PO_4_, 0.2 g/L of NaCl, 0.2 g/L of MgSO_4_, final pH of 7) [64] and VBMM [65]. When desired arginine and uracil were used at final concentrations of 40 mM and 10 mM, respectively.

### Bacterial growth assays

For evaluating growth, overnight cultures of each strain were prepared in liquid KB and incubated at 28 ° C with shaking. The next morning cultures were centrifuged and the pellets re-suspended in 1 mL of sterile water. The pellets were washed two more times and then re-suspended in 1 mL of sterile water. Following re-suspension, the OD_600_ of the cultures was measured, suspensions were diluted to OD_600_ = 2.0 in 1 mL of water, and the OD_600_ was measured again. The wells of a 96-well plate were filled with 200 μL of appropriate medium and then inoculated with 20 μL of bacterial suspension. Plates were incubated at 28.0 °C with shaking in a Biotek Synergy 2 microplate reader (Biotek, Winooski, VT). OD_600_ was measured every 30 min for 24 h. Three wells were measured for each bacterial strain/medium. When necessary, medium was supplemented with arginine (final concentration of 40 mM) and/or uracil (final concentration of 10 mM). Growth curves were repeated three times. Growth at time points 6, 12, 18 and 24 h was used for post-hoc statistical analysis. Statistical significance was assessed using Tukey HSD for pair-wise comparisons (α = 0.05).

### Apoplastic fluid extraction and growth

Apoplastic fluid was extracted from four-week old *Solanum lycopersicum* cv. MoneyMaker tomato plants following the protocol described in [53] with the following modifications. Whole leaves were removed and submerged in a container of DI water. The container was placed in a bell jar and a series of vacuum-pressure cycles were applied to the leaves at approximately 24 psi until the leaves were fully infiltrated. The leaves were removed, blotted dry, and carefully rolled into a 5-mL syringe barrel. The syringe was placed in a 15-mL conical vial and centrifuged at 2,000 rpm for 5 min at 4 °C to collect apoplastic fluid. Fluid was aliquoted into 1.5 mL microcentrifuge tubes and centrifuged again at 3,000 rpm for 10 min at 4 °C. The supernatant was removed and placed into a 1.5 mL microcentrifuge tube and stored at −80 °C. To test for cytoplasmic contamination, a fraction of the extracted apoplastic fluid was evaluated for Glucose-6-Phosphate Dehydrogenase (G6PDH) activity and compared to a leaf homogenate using a G6PDH Asssy Kit (Sigma-Aldrich, St. Louis, MO) according to the manufacturer’s instructions. Only apoplastic fluid that had little to no cytoplasmic contamination was used in growth analysis. Growth was evaluated using the protocol described above for bacterial growth assays. Three biological replicates were performed per strain, each with three technical replicates. Statistical significance was assessed using Tukey HSD (α = 0.05).

### Creation of reporter constructs

Genomic regions upstream of *carA* (PSPTO_4502) were amplified via PCR using chromosomal DNA isolated from wild-type DC3000. Primers 94 and 95 were designed to amplify the entire region between *dapB* (PSPTO_4503) and *carA* (PSPTO_4502) for a total product length of 228 bp. Primers 94 and 97 yielded a product of 187 bp in length that disrupted the putative stem loop region. Primers 94 and 98 amplified a region upstream of *P32* (143 bp). The 117 bp product obtained using primers 99 and 95 lacks the putative promoter upstream of *P32*. Primers 100 and 95 amplified a region of 51 bases upstream of *carA* that does not include *P32*. These amplified regions were cloned by PCR and TOPO cloning using the pENTR/D-TOPO vector (Invitrogen, Carlsbad CA). Positive clones were selected by plating on LB supplemented with 50 μg/ml of kanamycin. Inserts were then sequenced (Biotechnology Resource Center (BRC) at Cornell University) to identify correct clones. LR cloning and the Gateway® LR Clonase® II Enzyme mix (Invitrogen) were used to move the promoter regions into the destination vector pBS58, which contains a promoterless *lux* operon [66, 67]. The LR mixture was transformed into One Shot Omni-Mach 2 T1 cells (Invitrogen). Positive clones were selected by plating on LB supplemented with 50 μg/ml of kanamycin and 10 μg/ml of tetracycline and subsequently confirmed by sequencing.

### Promoter fusion assays

Promoter fusion constructs were introduced into the appropriate *P. syringae* strains using electroporation and plating transformants on KB plates containing kanamycin. Overnight cultures were prepared in KB medium supplemented with kanamycin and incubated at 28.0 °C with shaking then diluted the next day to an OD_600_ = 0.1 in VBMM or VBMM supplemented with 40 mM arginine and/or 10 mM uracil. 200 μL of the culture was dispensed into individual wells of a 96 well plate in a Biotek Syngery 2 microplate reader. The cultures were incubated at 28.0 °C with shaking. OD_600_ and relative luminescence were measured every 2 h and relative luminescence calculated as luminescence/OD_600_. The experiment was performed at least three times. Statistical significance was assessed using Tukey HSD (α = 0.05).

### RNA isolation

Total RNA was prepared using Trizol (Invitrogen) following the manufacturer’s instructions. Once isolated, RNA was treated with DNAse (Ambion, Austin, TX) to remove residual DNA. RNA was extracted using phenol:chloroform: IAA (isoamyl alcohol) then cleaned and concentrated using RNA Clean-up & Concentrator kit (Zymo Research, Irvine, CA). Removal of DNA was verified by quantitative real-time PCR with primers to the normalizing genes *gap 1* (PSPTO_1287) or *gyrA* (PSPTO_1745) [68].

### Reverse transcription-PCR (RT-PCR)

Total RNA (100 ng) was reverse transcribed using Superscript III (Invitrogen) and primers listed in Additional file 2: Table S1 according to the manufacturer’s instructions. PCR reactions were performed for 30 cycles. The PCR products were separated by agarose gel electrophoresis.

### 3′ rapid amplification of cDNA ends (RACE)

3′ RACE was performed as described by Moll et al. [69]. This protocol was adapted from Argaman et al. [70].

### Quantitative real-time PCR (qPCR)

qPCR was performed as described by Park *et al.* [71]. Extracted RNA was synthesized into cDNA using the qScript cDNA Supermix (Quanta Biosciences, Gaithersburg, MD) and qPCR was performed using IQ SYBR green Supermix (Bio-Rad, Hercules, CA) on a iQ5 multicolor real-time detection system (BioRad). The production of nonspecific products was determined by the dissociation protocol included in the software provided with the machine. All primer pairs were found to yield unique products using the dissociation protocol (data not shown). The PCR assay was carried out as previously described [71]. Gene expression fold-change was calculated using the ΔΔ C_t_ method. C_t_ values of each gene tested were normalized to the C_t_ values of the housekeeping gene *gap1* (PSPTO_1287). Primers used for qRT-PCR are listed in Additional file 2: Table S1.

### Construction of mutant strains

Primers used for the construction of mutant strains are listed in Additional file 2: Table S1. Unmarked deletion strains were constructed using pK18mobsacB plasmid [72]. DNA fragments of approximately 1.0 kb upstream and downstream of *P32*, *carA*, and *argR* were amplified by PCR, gel purified and then joined by splicing by overlap extension PCR. The *P32*, *carA*, and *argR* genes were then deleted from DC3000 using the deletion constructs and marker exchange mutagenesis [71]. Mutant clones (those containing the deletion) were confirmed by DNA sequencing.

### Complementation of *ΔcarA*

The coding region of *carA* along with its native Shine Dalgarno sequence was amplified from DC3000 genomic DNA using oligos SCMF3 F and SCMF4 R and the Expand High Fidelity PCR System from Roche. The primers contained the restriction enzyme site XbaI at their 5′ ends. The XbaI-digested PCR product was cloned into the XbaI site of broad host range vector pUCP22 containing the lac promoter [73], and sequenced to confirm the presence of *carA*. The resulting plasmid was designated as pUCP22::*carA*.pUCP22::*carA* was electroporated into DC3000*ΔcarA* to generate the complementation strain of *ΔcarA*. For controls, pUCP22 was electroporated into DC3000 and *ΔcarA*. The strains were selected on gentamycin at 5 μg/ml. Bacterial growth assays were performed as described above.

### Evaluating virulence in Arabidopsis plant seedlings

To assess virulence, the *Arabidopsis* seedling flood-inoculation assay was used [74] following the modifications described in Park *et al*. [71]*.*

### Tomato Dip-inoculation

Tomato dip inoculations were performed as described by Park *et al.* [71].

### Motility assays

*P. syringae* strains were grown overnight at 28 °C in KB. Overnight cultures were diluted to OD_600_ of ~0.3 and 5 μl were used to spot onto swarming plates or stab onto swimming plates. Swarming plates consisted of nutrient broth (8 g/L) and 0.5 % (wt/vol) agar. Swimming assays were performed using nutrient broth (8 g/L) and 0.3 % (wt/vol) agar. Swarm and swim zones were measured after plates were incubated for 24 h at room temperature. Three technical replicates were performed for each experiment and each experiment was performed three times. Data were analyzed by one-way analysis of variance (ANOVA) followed by Tukey HSD for pair-wise comparisons.

### Biofilm formation

*P. syringae* strains were grown overnight at 28 °C in KB. Overnight cultures were washed three times with nutrient broth and diluted to OD_600_ of 1.0. Cultures were added to 96-well plates pre-filled with media to final OD_600_ of 0.1 and allowed to incubate at 28 °C for 72 h under static conditions. After 72 h of incubation the OD_600_ was measured and media was removed from each well. Biofilm formation was assessed based on protocols described by Merritt et al. [75] and O’Toole et al. [76]*.* Approximately 250 μl of 0.1 % crystal violet stain was added to each well and allowed to incubate for 5 min. The stain was removed and wells were washed three times with ddH_2_0. The stained biofilms were resuspended in 30 % acetic acid and OD_570_ was recorded for each well. Four replicates of each strain were normalized using the final OD_600_, averaged, and standard deviation was computed. Statistical significance was assessed using a one-way ANOVA test followed by Tukey HSD for pair-wise comparisons.

## Additional files

Additional file 1: Figure S1. Expression of *carA* in the *P32* mutant. **Figure S2** Growth of the complemented mutant of *ΔcarA* is comparable to wild type DC3000. (PDF 167 kb) Additional file 2: Table S1. List of plasmids, strains, and primers. (PDF 96 kb)

## Abbreviations

CFU: Colony forming units
CPSase: Carbamoylphosphate synthetase
FLOE: Fluorescently labeled oligonucleotide extension
G6PDH: Glucose-6-phosphate dehydrogenase
HR: Hypersensitive response
IVET: In vivo expression technology
KB: King’s B
MG: Mannitol-glutamate
qPCR: Quantitative real-time PCR
RACE: Rapid amplification of cDNA ends
T3SS: The type III secretions system
VBMM: Vogel-bonner minimal medium
