# Supplementary material for: Disruption of the carA gene in Pseudomonas syringae results in reduced fitness and alters motility
Source: BMC Microbiol. 2016 Aug 24;16(1):194. doi: 10.1186/s12866-016-0819-z (PMC4997734; doi:10.1186/s12866-016-0819-z)
Supplement: Additional file 1: Figure S1. — Expression of carA in the P32 mutant. Figure S2 Growth of the complemented mutant of ΔcarA is comparable to wild type DC3000. (PDF 167 kb) [file 12866_2016_819_MOESM1_ESM.pdf]

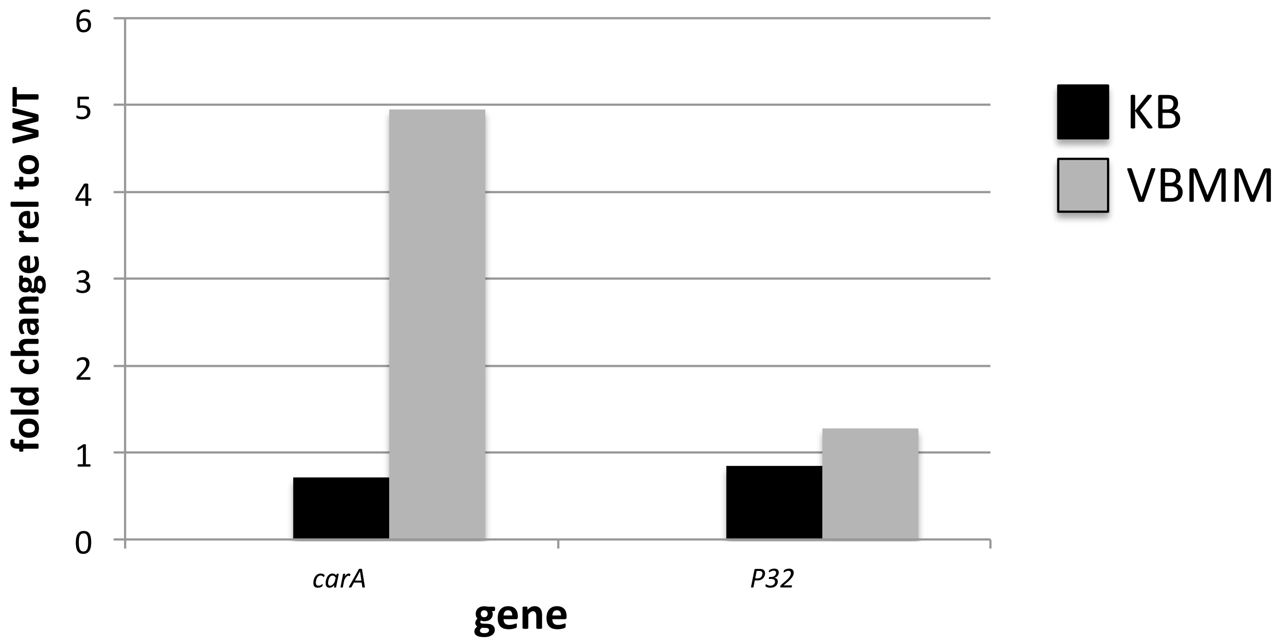

Fig. S1. Expression of *carA* in *ΔP32* mutant. Transcript levels of *carA* and *P32* in *ΔP32* mutant were compared using qRT-PCR. The black bars represent the ratios of the transcripts comparing *ΔP32* mutant to the WT in KB medium and the gray bars represent the ratios of the transcripts comparing *ΔP32* mutant to the WT in VBMM. RNA samples were normalized using *gap1*. Results show that the carA gene is expressed in a *ΔP32* mutant when the cells are grown in the minimal medium VBMM.

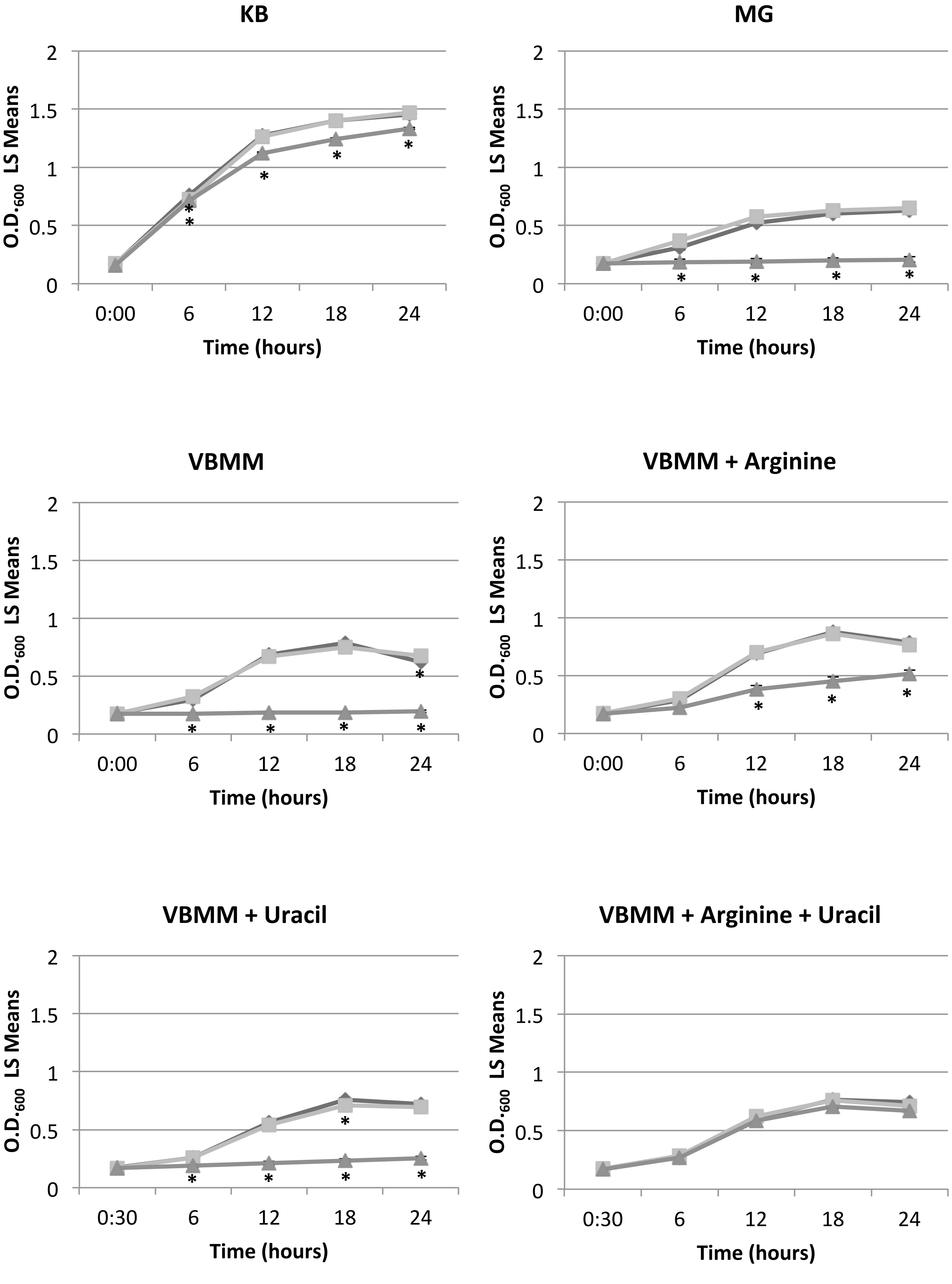
KB
MG
VBMM
VBMM + Arginine
VBMM + Uracil
VBMM + Arginine + Uracil
O.D.600 LS Means
0
0.5
1
1.5
2
Time (hours)
0:00
0:30
6
12
18
24
*

Figure S2. Growth of the complemented mutant of *ΔcarA* is comparable to wild type DC3000. Growth of wild type DC3000 (pUCP22) (dark gray diamonds), *ΔcarA* (pUCP22:*carA*) (light gray squares) and *ΔcarA* (pUCP22) (light grey triangles) in KB, MG, VBMM, VBMM supplemented with 40 mM arginine, VBMM supplemented with 10 mM uracil, and VBMM supplemented with 40 mM arginine and 10 mM uracil. Growth is represented as least squares means (LS Means) with standard error of $O.D._{600}$ over time. The data shown represent 4 biological replicates per strain, each with 3 technical replicates. Post hoc comparisons were performed using Tukey HSD (α=0.05). For each time point, the values which are significantly different from wild-type are shown with an asterisk. Statistical analyses were performed using JMP Pro 11.
