# Supplementary material for: Disruption of the carA gene in Pseudomonas syringae results in reduced fitness and alters motility
Source: BMC Microbiol. 2016 Aug 24;16(1):194. doi: 10.1186/s12866-016-0819-z (PMC4997734; doi:10.1186/s12866-016-0819-z)
Supplement: Additional file 2: Table S1. — List of plasmids, strains, and primers. (PDF 96 kb) [file 12866_2016_819_MOESM2_ESM.pdf]

Primer	Sequence
oSWC06433	GCGAAGCCATTGGAGCCGA
oSWC06434	CGATGCCAGCGATGGCAA
oSWC06435	CGGCAGGTCACGGATGACCAGA
oSWC06439	GCAAAAAAGCGGAATGACGTAGTTA
oBGB00034	CAGGCTCATTGGTGGTAGACCAAA
oBGB00035	CCACTAAAAGCGCGCAGATGATT
oBGB00036	GCAAAAAAGCGGAATGACGTAGTTA
oBGB00066	GCTCTAGAGTCGATCGTCCCGACAGCA
oBGB00067	CGCTTTTAGTGGACACACTAAAGCTGTA
oBGB00068	*GCTTTAGTGTGTCCACTAAAAGCG*GCAACCTGCGATCG CCCT
oBGB00069	GCTCTAGACGTCGAACAGCGACTTGTGGATA
oBGB00070	GGCTTAGTCAAGAAGACCTCCCGTA
oBGB00071	*GGGAGGTCTTCTTGACTAAGCC*CGCTGACCGGTTGCCT TGA
oBGB00072	GCTCTAGAGGACAGCTCGTCGAGGGTGTAA
oBGB00087	GTCCATCGGCCTGGCGTG
oBGB00088	TCGAAGAGTTCCGCACATCG
oBGB00089	CCGCAGGTCAGTCGGCAAG
oBGB00090	CAGCTTCGGATCAAGGCCAC
oBGB00091	GCGTCAAGGTCAACATCC
oBGB00092	TTGTAGGCAAGCACTTCG
oBGB00093	GTCGCAGCGATGGCCTCT
oBGB00094	CACCACGACATGCAGGATGTACTGGG
oBGB00095	CAAGAAGACCTCCCGTAAATAAAGCC
oBGB00096	TGCAAAAAAGCGGAATGACGTAG
oBGB00097	GTTACGTCACCCCGCTTTCTTATG
oBGB00098	GTGGACACACTAAAGCTGTAGCTTACAG
oBGB00099	CACCACTAAAAGCGCGCAGATGATTCC
oBGB00100	CACCCAACCTGCGATCGCCCTTT
oBGB00112	GCTCTAGATGGCGTGGTGACATTCCTGC
oBGB00113	GGAACATCCAGGTCATGTCACG
oBGB00114	CGTGACATGACCTGGATGTTCCCGAGCGCGGTTGATT GTCTTG
oBGB00115	GCTCTAGAGTGCTCGTCACACAGTTTGCG

SCMF3 F	GCGC-TCTAGA-TGCAACCTGCGATCGCCCTT
SCMF4 R	GCGC-TCTAGA-TCAGCGTCGCTTGGCCATGG

Description
carA forward (5' race)
carA GSP1 5' race
carA GSP2 5' race
P32 (upstream of carA) GSP1
upstream of P32 fwd
P32 fwd
P32 (upstream of carA) GSP1
ncr0186 up fwd (incl. XbaI)
ncr0186 up rev
ncr0186 do fwd (incl. tail compl. to #67)
ncr0186 do rev (incl. XbaI)
carA up rev
carA do fwd (incl. tail compl. to #70)
carA do rev (incl. XbaI)
carA seq fwd
ncr0186 and carA seq for
ncr0186 seq rev
carA seq rev
carA sense for RT-PCR
carA antisense for RT-PCR
crc do seq rev (outside of cloned region)
carA-lux-fwd1 (WT carA for lux fusion)
carA-lux-rev1 (WT for lux fusion)
carA-lux-rev2 (lacking region between P32 and carA)
carA-lux-rev3 (lacking half of stem loop)
carA-lux-rev4 (only upstream of P32)
carA-lux-fwd2 (no promoter upstream of P32)
carA-lux-fwd3 (region between P32 and carA)
argR up for (XbaI)
argR up rev
argR do fwd (incl compl tail)
argR do rev (incl. XbaI)

F primer to amplify the coding region of carA along with the native Shine Dalgarno sequence from DC3000; contains restriction site XbaI at the 5’ end
R primer to amplify the coding region of carA from DC3000; contains restriction site XbaI at the 5’ end

Strain/plasmid	Relevant Characteristics	Source
**<u>Plasmids</u>**		
pENTR/D	Topo cloning Gateway entry vector	Invitrogen
pK18mobsacB	pMB1, *mob*, *sacB*, sucrose^S, Kan^R	[23]
pBB67	pK18mobsacB-Δncr0186; Kan^R	This work
pBB68	pK18mobsacB-ΔcarA; Kan^R	This work
pBB73	pENTR/D containing *carA* promoter region P1); Kan^R	This work
pBB74	pENTR/D containing *carA* promoter region P3; Kan^R	This work
pBB75	pENTR/D containing *carA* promoter region P4; Kan^R	This work
pBB76	pENTR/D containing *carA* promoter region P5; Kan^R	This work
pBB77	pENTR/D containing *carA* promoter region P6; Kan^R	This work
pBS208	*lux* reporter construct	(Markel *et al.*, 2011; Swingle *et al.*, 2008)
pBB78	*lux* reporter construct containing *carA* promoter region P1; Kan^R, Tet^R	This work
pBB79	*lux* reporter construct containing *carA* promoter region P3; Kan^R, Tet^R	This work
pBB80	*lux* reporter construct containing *carA* promoter region P4; Kan^R, Tet^R	This work
pBB81	*lux* reporter construct containing *carA* promoter region P5; Kan^R, Tet^R	This work
pBB82	*lux* reporter construct containing *carA* promoter region P6; Kan^R, Tet^R	This work
pBB83	pK18mobsacB-*ΔargR*; Kan^R	This work
pUCP22	Broad host range plasmid with *lac* promoter region, Gent ^{R}	(West, 1984)
pUCP22::*carA*	Coding region of *carA* cloned downstream to *lac* promoter in pUCP22, Gent ^{R}	This work
**<u>Strains</u>**		
DC3000 (WT)	*P. syringae pv. tomato* DC3000 wild-type strain DC3000, rif^R	(Buell, 2003)
E. coli (pBB67)	DH5α containing pK18mobsacB-Δncr0186; Kan^R	This work
E. coli (pBB68)	DH5α containing pK18mobsacB-ΔcarA; Kan^R	This work

E. coli (pBB73)	Mach 1-TR1	This work
E. coli (pBB74)	Mach 1-TR1	This work
E. coli (pBB75)	Mach 1-TR1	This work
E. coli (pBB76)	Mach 1-TR1	This work
E. coli (pBB77)	Mach 1-TR1	This work
E. coli (pBB78)	Top10	This work
E. coli (pBB79)	pBB79	This work
E. coli (pBB80)	pBB80	This work
E. coli (pBB81)	pBB81	This work
E. coli (pBB82)	pBB82	This work
DC3000 (pBB78)	Wild-type strain with pBB78	This work
DC3000 (pBB79)	Wild-type strain with pBB79	This work
DC3000 (pBB80)	Wild-type strain with pBB80	This work
DC3000 (pBB81)	Wild-type strain with pBB81	This work
DC3000 (pBB82)	Wild-type strain with pBB82	This work
Δ*carA*	Δ*carA* deletion DC3000 mutant	This work
Δ*P32*	Δ*P32* deletion DC3000 mutant	This work
Δ*argR*	Δ*argR* deletion DC3000 mutant	This work
